# Evolution of interdisciplinarity in biodiversity science

**DOI:** 10.1002/ece3.5244

**Published:** 2019-06-10

**Authors:** Dylan Craven, Marten Winter, Konstantin Hotzel, Jitendra Gaikwad, Nico Eisenhauer, Martin Hohmuth, Birgitta König‐Ries, Christian Wirth

**Affiliations:** ^1^ Biodiversity Macroecology & Biogeography Faculty of Forest Sciences and Forest Ecology University of Göttingen Göttingen Germany; ^2^ German Centre for Integrative Biodiversity Research (iDiv) Halle‐Jena‐Leipzig Leipzig Germany; ^3^ qouch GbR Jena Germany; ^4^ Institute of Computer Science Friedrich Schiller University Jena Jena Germany; ^5^ Institute of Biology Leipzig University Leipzig Germany; ^6^ Max‐Planck Institute for Biogeochemistry Jena Germany

**Keywords:** bibliographic analysis, biodiversity, conceptual homogenization, interdisciplinarity, topic models

## Abstract

The study of biodiversity has grown exponentially in the last thirty years in response to demands for greater understanding of the function and importance of Earth's biodiversity and finding solutions to conserve it. Here, we test the hypothesis that biodiversity science has become more interdisciplinary over time. To do so, we analyze 97,945 peer‐reviewed articles over a twenty‐two‐year time period (1990–2012) with a continuous time dynamic model, which classifies articles into concepts (i.e., topics and ideas) based on word co‐occurrences. Using the model output, we then quantify different aspects of interdisciplinarity: concept diversity, that is, the diversity of topics and ideas across subdisciplines in biodiversity science, subdiscipline diversity, that is, the diversity of subdisciplines across concepts, and network structure, which captures interactions between concepts and subdisciplines. We found that, on average, concept and subdiscipline diversity in biodiversity science were either stable or declining, patterns which were driven by the persistence of rare concepts and subdisciplines and a decline in the diversity of common concepts and subdisciplines, respectively. Moreover, our results provide evidence that conceptual homogenization, that is, decreases in temporal β concept diversity, underlies the observed trends in interdisciplinarity. Together, our results reveal that biodiversity science is undergoing a dynamic phase as a scientific discipline that is consolidating around a core set of concepts. Our results suggest that progress toward addressing the biodiversity crisis via greater interdisciplinarity during the study period may have been slowed by extrinsic factors, such as the failure to invest in research spanning across concepts and disciplines. However, recent initiatives such as the Intergovernmental Science‐Policy Platform on Biodiversity and Ecosystem Services (IPBES) may attract broader support for biodiversity‐related issues and hence interdisciplinary approaches to address scientific, political, and societal challenges in the coming years.

## 1 INTRODUCTION

Conserving biodiversity came to prominence as a global issue toward the end of the 20th century amid growing concerns for biodiversity change (Butchart et al., 2010; Pereira et al., 2010). Drivers of biodiversity loss are multiple (Maxwell, Fuller, Brooks, & Watson, 2016), as are its consequences for human livelihoods (Cardinale et al., 2012). A prerequisite for tackling this crisis with appropriate policy measures is a fundamental understanding of biodiversity: How much there is, why does it occur, where does it occur, how does it work, and what benefits does it generate? The search for answers to these fundamental questions in biodiversity can be traced to major scientific achievements in biology, such as Darwin's theory of evolution (Darwin, 1859), Mendel's rules of heredity (Mendel, 1865), Hutchinson's concept of the ecological niche (Hutchinson, 1959), and Wilson and MacArthur's theory of island biogeography (MacArthur & Wilson, 1967).

In recent decades, biodiversity science (United Nations, 1992)—a field of study spanning a variety of related and well‐defined subdisciplines—has expanded greatly in size, that is, number of publications, and in breadth, that is, range of subdisciplines (Loreau, 2010). Underlying this growth is an array of complex scientific problems centered around the description and prediction of biodiversity patterns and processes using data from genes, individuals, communities, and ecosystems across temporal and spatial scales (Chase et al., 2018; Price & Schmitz, 2016) as well as their implications for and linkages to other global political and societal challenges, such as climate change, human health, and poverty (Adams et al., 2004; Barnosky et al., 2012; Cardinale et al., 2012; Civitello et al., 2015). Major technological advances in computing power, remote sensing, and omics have provided new tools to improve understanding of biodiversity patterns and where and how to conserve it, yet significant gaps and biases in biodiversity data may limit our ability to do so (Hortal et al., 2015). Despite appeals for interdisciplinary approaches in biodiversity science to meet scientific, political, and societal challenges (Liu et al., 2007), numerous barriers, such as communication difficulties and institutional barriers, may prevent the adoption of interdisciplinary approaches (Roy et al., 2013).

**Figure 1:**
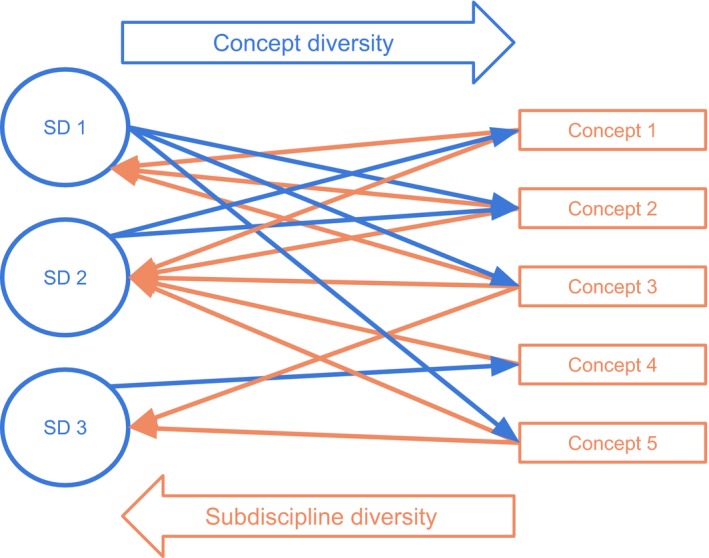
Illustration of interdisciplinarity that represents concept diversity across subdisciplines (blue arrows) and subdiscipline diversity across concepts (orange arrows), respectively. *SD* indicates subdiscipline (classified by Thomson Reuters), and concept (classified by a topic model) indicates a topic or an idea. The direction of the arrows indicates “usage.” For example, blue arrows pointing toward orange arrows shows that subdisciplines are drawing upon the concepts to which they are connected. In this framework, “SD1” would be the most diverse and conceptually heterogeneous subdiscipline (as indicated by the blue arrows connecting it to three concepts), and “SD3” would be the least diverse and most conceptually homogeneous subdiscipline (as indicated by the blue arrow connecting it to only one concept), while “Concept 3” would be the most interdisciplinary concept (as indicated by the orange arrows connecting it to three subdisciplines), and “Concept 4” would be the least interdisciplinary concept (as indicated by the orange arrow connecting it to only one subdiscipline)

Interest in interdisciplinary research and the quantification of interdisciplinarity across scientific disciplines has grown considerably in the past 30 years (Morillo, Bordons, & Gómez, 2003; Porter & Rafols, 2009; Wang et al., 2017). Increasingly, academic institutions and funding agencies have recognized that solutions to complex problems frequently lie at the boundary between disciplines (National Academy of Sciences, 2004), and there is evidence that certain well‐established fields of research, for example, physics, mathematics, and medicine, are becoming more interdisciplinary (Larivière & Gingras, 2010; Pan, Sinha, Kaski, & Saramäki, 2012; Porter & Rafols, 2009). Therefore, we examined whether biodiversity science has responded to a growing array of challenges by bridging traditional gaps among its subdisciplines via an increase in interdisciplinarity, here quantified as the diversity of concepts and subdisciplines and interactions between concepts and subdisciplines. We defined concept diversity as the diversity of topics and ideas (hereon referred to as concepts) across subdisciplines and subdiscipline diversity as the diversity of subdisciplines associated with particular concepts (Figure 1; Porter & Rafols, 2009; Rafols & Meyer, 2010); we considered interactions among concepts and subdisciplines to occur when an article within a given subdiscipline used a particular concept. Using a comprehensive data set of published articles on concepts associated with biodiversity in natural and social sciences from 1990 to 2012, we asked whether biodiversity science has become more interdisciplinary by using (a) a more diverse array of concepts, (b) an increasing variety of subdisciplines, and/or (c) because interactions between concepts and subdisciplines have become less specialized.

## 2 METHODS

Until recently, patterns of interdisciplinarity have been analyzed predominantly with coarse approximations of how concepts are used among and within subdisciplines, for example, the number of citations among (or within) subdisciplines, journals, or authors (Cassi, Champeimont, Mescheba, & Turckheim, 2017; Pan et al., 2012; Vaz et al., 2017). However, such an approach relies upon classifying articles into broad categories (e.g., PACS codes in physics; Pan et al., 2012), which assumes that all articles in a given category are conceptually similar and cannot provide further insight about whether and how articles within a particular subdiscipline are related. More granular approaches, such as topic models and automated content analysis (Blei, Ng, & Jordan, 2003; McCallen et al.,^2019^), analyze word co‐occurrence patterns within articles and classify them into similar concepts to explore patterns of interdisciplinarity. Here, we fit a topic model (see Section 2.3) using the text of published articles in biodiversity science to group articles into concepts. We then analyze the model results using commonly used ecological tools, for example, diversity indices and bipartite networks, to address our research questions.

### 2.1 Data collection

We compiled a data set comprised of publications in peer‐reviewed journals by querying the Web of Knowledge using the following terms: “biodiv*,” “species diversity,” “functional diversity,” “ecosystem diversity,” and “genetic diversity.” These query terms reflect a broad definition of biodiversity recognized by the international scientific community (United Nations, 1992). To reduce misidentification of articles, we restricted our query to subdisciplines in the natural and social sciences likely to be related to biodiversity (Supplementary Note). We extracted the title, abstract, year of publication, and subdisciplines for each article and eliminated articles that did not have abstracts. We used the Web of Science schema for categorizing journals to subdisciplines (“research areas”), which assigns each journal to one or more subject categories and includes subfields within broader disciplines (Thomson Reuters, 2016). In total, our data set is comprised of 97,945 articles published from 1990 to 2012.

### 2.2 Data preparation (natural language processing)

We extracted terms from the title and abstract of each article in our data set using natural language processing tools with the Apache Hadoop software library (Hadoop, 2019). We divided text into sentences and then words, which were subsequently classified into parts of speech and grouped different forms of words into a single term. Terms included as stop words were labeled and excluded from subsequent analyses. Prior to fitting topic models, we calculated the inverse document frequency (idf) for each term across all articles in the data set and removed terms from the vocabulary that occurred infrequently (see Supporting information). In doing so, we reduced the number of terms in our vocabulary, which has been shown to improve the precision of text classification (Jing, Huang, & Shi, 2002). In total, 2,552 terms formed the vocabulary used in all analyses.

### 2.3 Continuous time dynamic topic model (LDA‐cDTM)

We used a continuous time dynamic topic model (LDA‐cDTM) to classify each article to an underlying set of topics (Figure S1; Blei et al., 2003; Wang, Blei, & Heckerman, 2012). LDA‐cDTM is a hierarchical Bayesian model that uses Brownian motion to model topics over time for a sequential collection of documents and use the variational Kalman filter to estimate the posterior distributions of topic structure (Wang et al., 2012). Topics, hereon referred to as concepts, are latent variables representing abstract ideas that are comprised of a set of co‐occurring terms that shift over time. LDA‐cDTMs identify each concept as a distribution over terms and use prior distributions of previous time steps to inform how the model delineates concepts in subsequent time steps. As LDA‐cDTMs use a latent variable approach to define each concept, the delineation of a concept is not dependent upon the presence of a particular term, thus allowing terms to be added or removed (Wang et al., 2012). For example, a concept related to secondary succession might have a high frequency of terms like “forest,” “grassland,” “diversity,” and “composition” across the entire time period. These terms may initially co‐occur with terms such as “species,” “r‐strategy,” and “k‐strategy” and later with terms such as “functional,” “trait,” and “phylogenetic.” While this concept always describes changes in diversity and composition during succession, the turnover in terms may reflect how the focus of this concept has evolved from a species‐based approach (Bazzaz, 1979) to one that emphasizes phylogenetic and functional trait information (Letcher, 2010; Raevel, Violle, & Munoz, 2012).

We divided our data set into four continuous time steps of approximately five years each: 1990–1995, 1996–2000, 2001–2005, and 2006–2012. We used five‐year time steps because we expected concepts in biodiversity science to evolve gradually and to ensure that model results were interpretable. When fitting LDA‐cDTMs using shorter time steps, we found that the resulting concepts were difficult to interpret with the associated terms (results not presented).

We fitted LDA‐cDTMs using a range of numbers of concepts (1–150) to our data set and calculated two model fit parameters, log‐likelihood and perplexity (Blei et al., 2003), to identify the most parsimonious model (Figure S2). We selected the model with 50 concepts as this represented the break point in both parameters, that is, where information gain of models containing more concepts began to reach an asymptote. This model assigned the most probable concepts to each article in our data set, which we used for subsequent analyses (see below) and for each term calculated the probability (φ_kw_) that it would occur in the assigned concepts. Terms and subdisciplines associated with each concept can be examined interactively online (http://data.idiv.de/repo/Accelerating_interdisciplinarity_in_biodiversity_sciences/).

### 2.4 Data analysis

To examine patterns of interdisciplinarity in biodiversity science, we used analytical methods commonly used in biodiversity science and bibliometrics: diversity indices and network structure (Rafols & Meyer, 2010; Stirling, 2007; Wagner et al., 2011). In the case of diversity indices, we treated subdisciplines as ecological communities and concepts as species (or vice versa), while for network analysis, we treated subdisciplines and concepts as separate trophic groups. We used both methods because they provide complementary insights into interdisciplinarity; diversity indices can reveal the number of concepts and their evenness within subdisciplines (and vice versa), while measures of network structure show patterns of interactions between concepts and subdisciplines.

#### 2.4.1 Concept and subdiscipline diversity

We estimated concept and subdiscipline diversity by calculating species richness and the effective number of species for the probability of interspecific encounter (ENS_PIE_), which is equivalent to 1/Simpson's index, for each time step using the “mobr” package in R (McGlinn et al.,^2019^). Species richness gives emphasis to the contributions of rare concepts or subdisciplines, while ENS_PIE _gives greater weight to the highly abundant concepts or subdisciplines. For ENS_PIE_, abundance is the number of articles within each concept or within each subdiscipline. We additionally calculated rarefied species richness (*n* = 25 articles) for both concepts and subdisciplines, to account for differences in abundance.

We then identified concepts that had become more or less interdisciplinary over time, by calculating relative change between 1990 and 2012 in subdiscipline diversity for species richness and ENS_PIE_. Concepts in the upper 10% quantile were classified as “increasing,” while those in the lower 10% quantile were classified as “decreasing,” and those whose relative change was between the upper and lower 10% quantiles were classified as “stable.” In the main text, we focus on concepts classified based on ENS_PIE_; certain concepts could not be classified into the above‐mentioned groups because ENS_PIE_ could not be estimated reliably, that is, where the number of articles was the same as the number of concepts or subdisciplines (McGlinn et al.,^2019^).

We assessed temporal changes in the composition between 1990 and 2012 within subdisciplines and within concepts by calculating temporal *β* diversity and its components, gains and losses, using Jaccard or Ruzicka dissimilarity with the “TBI” function in R (Winegardner, Legendre, Beisner, & Gregory‐Eaves, 2017). Jaccard dissimilarity gives equal weight to the contribution of rare concepts or subdisciplines, while Ruzicka dissimilarity uses abundances and emphasizes common concepts or subdisciplines (Legendre, 2014). Gains and losses of concepts (or subdisciplines) indicate which process dominates the observed shifts in composition within a subdiscipline (or within a concept).

#### 2.4.2 Interactions among subdisciplines and concepts

To examine changes in interactions among subdisciplines and concepts, we first built bipartite networks using cross‐citation information, where concepts and subdisciplines represent different trophic levels and each link weight represents the number of articles that is classified by the LDA‐cDTM to belong to a given concept within a given subdiscipline. We then calculated niche overlap using bipartite networks for each time step with the R package “bipartite” (Dormann, Gruber, & Fruend, 2008). Niche overlap is the mean similarity of interaction patterns within a trophic group and quantifies the extent to which subdisciplines use concepts and vice versa (Dormann et al., 2008); higher values indicate that concepts use similar subdisciplines or that subdisciplines use similar concepts. We weighted niche overlap using link weights, as unweighted measures may be biased by sample size (Tylianakis, Tscharntke, & Lewis, 2007). We also fit null models to test whether observed values differed than one would expect at random using the *null.t.test* function and 1,000 null models.

We calculated 95% confidence intervals using 1,000 bootstrapped replicate samples using the R package “rms” (Harrell, 2017) and interpret differences across time steps as significant if confidence intervals do not overlap. Unless mentioned otherwise, all analyses were performed with R 3.4.4 (R Core Team 2018).

## 3 RESULTS

**Figure 2:**
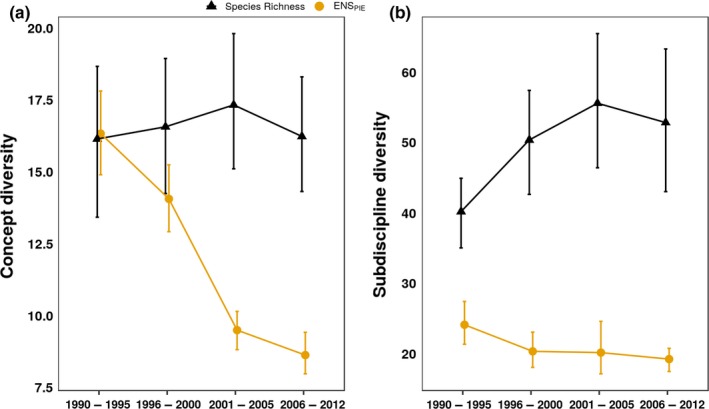
Temporal changes in mean concept diversity (a) and mean subdiscipline diversity (b) in biodiversity science between 1990 and 2012. Concept diversity and subdiscipline diversity were estimated using species richness and the effective number of species for the probability of interspecific encounter (ENS_PIE_), respectively. The former highlights contributions of rare species, while the latter emphasizes contributions by dominant or highly abundant species. Concept diversity is the number of concepts (identified by a continuous time dynamic topic model) associated with a subdiscipline, and subdiscipline diversity is the number of subdisciplines associated with a concept (see Figure 1). For ENS_PIE_, abundance is the number of articles in a concept or subdiscipline. Whisker bars are bootstrapped 95% confidence intervals. For terms and biodiversity subdisciplines associated with each concept in each time step, follow this link: (http://data.idiv.de/repo/Accelerating_interdisciplinarity_in_biodiversity_sciences/)

To test whether interdisciplinarity in biodiversity science increased, we examined changes in concept and subdiscipline diversity over the past twenty years. We found that concept and subdiscipline diversity were stable from 1990 to 2012 (species richness; Figure 2), as were rarefied concept and subdiscipline diversity (Figure S3). However, when weighted by abundance (ENS_PIE_), both concept and subdiscipline diversity decreased significantly over time (Figure 2; 95% confidence intervals did not overlap). The contrast in temporal trends of species richness and ENS_PIE_ for both concept and subdiscipline diversity reveals that rare concepts and subdisciplines contributed disproportionately to maintaining interdisciplinarity over the study period.

**Figure 3:**
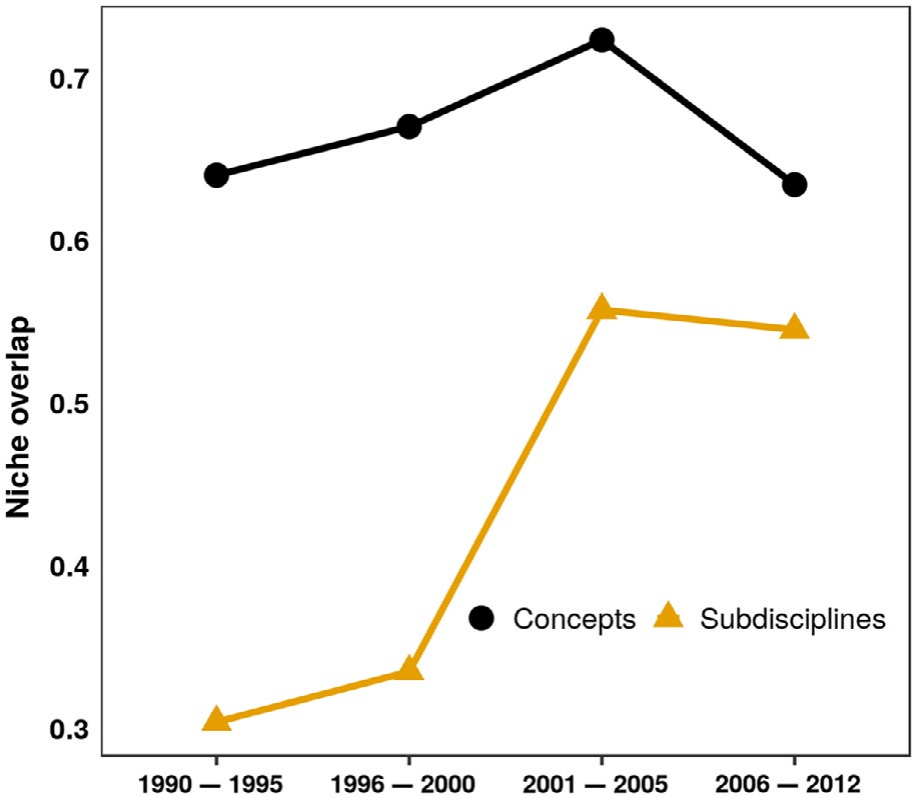
Temporal changes in niche overlap for subdisciplines and concepts in biodiversity science between 1990 and 2012. Niche overlap represents the extent to which subdisciplines use similar concepts and vice versa; higher values indicate greater similarity in the interaction pattern among concepts (or subdisciplines). Concepts are “topics” identified by a continuous time dynamic topic model

We then assessed temporal patterns in interactions among subdisciplines and concepts in biodiversity science. Our results show that niche overlap of subdisciplines was consistently lower than that of concepts from 1990 to 2012 (Figure 3). However, niche overlap of subdisciplines increased strongly over time, while that of concepts was consistent over the study period (Figure 3). In all time periods, observed values of niche overlap differed significantly to what would be expected at random (*p* > 0.001).

**Figure 4:**
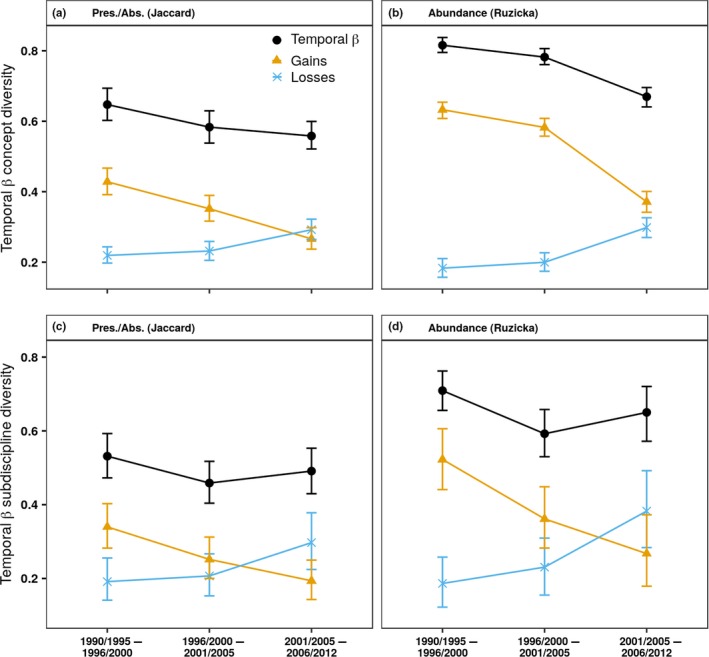
Temporal changes in the composition of concepts within subdisciplines (a, b) and concepts (c, d) in biodiversity science between 1990 and 2012. Temporal *β* diversity was estimated using Jaccard (a, c; presence/absence) or Ruzicka dissimilarity (b, d; abundance weighted) and was partitioned into gains and losses of concepts within a subdiscipline (temporal *β* concept diversity) or of subdisciplines within a concept (temporal *β* subdiscipline diversity). Abundance is the number of articles in a concept or subdiscipline; concepts were identified by a continuous time dynamic topic model. Whisker bars are bootstrapped 95% confidence intervals

To further disentangle the temporal patterns of concept and subdiscipline diversity, we evaluated temporal changes in composition within biodiversity subdisciplines and concepts. We found that dissimilarity in composition over time within subdisciplines, calculated as temporal *β* diversity, decreased significantly with time when calculated using either Jaccard or Ruzicka dissimilarity (Figure 4a, b; 95% confidence intervals do not overlap), by 13.7% and 17.9%, respectively. This pattern was driven by two simultaneous processes: (a) a significant decline in the accumulation of new concepts within biodiversity subdisciplines over time (“Gains”; Figure 4a, b) and (b) a significant increase in the loss of old concepts over time (“Losses”; Figure 4a, b). In contrast, temporal β subdiscipline diversity did not vary significantly over time for either Jaccard or Ruzicka dissimilarity (Figure 4c, d), declining by 7.5% and 8.3%, respectively. The overall pattern obscured significant declines in the accumulation of new subdisciplines within concepts and significant increases in the loss of old subdisciplines within concepts (Figure 4c, d).

**Figure 5:**
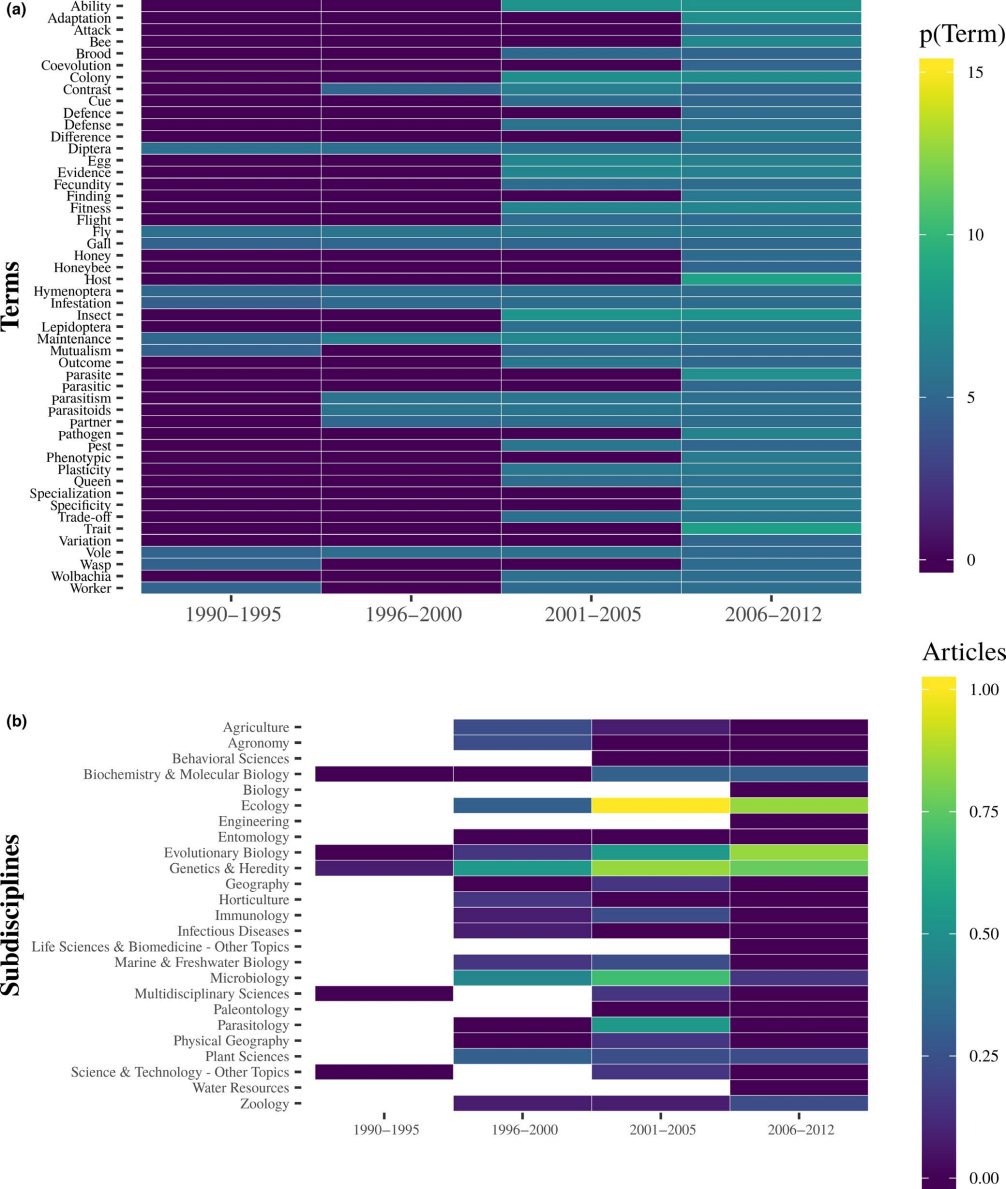
Temporal change in (a) the probability of highly ranked terms and (b) abundance of subdisciplines associated with “Concept 8,” a concept that addresses disease resistance in social insects and that became more interdisciplinary from 1990 to 2012. The top 50 terms from 2006 to 2012 (ranked by p(Term)) were selected. The top 25 ranked subdisciplines from 2006 to 2012 were selected, and the number of articles was scaled to facilitate comparisons with other concepts

**Figure 6:**
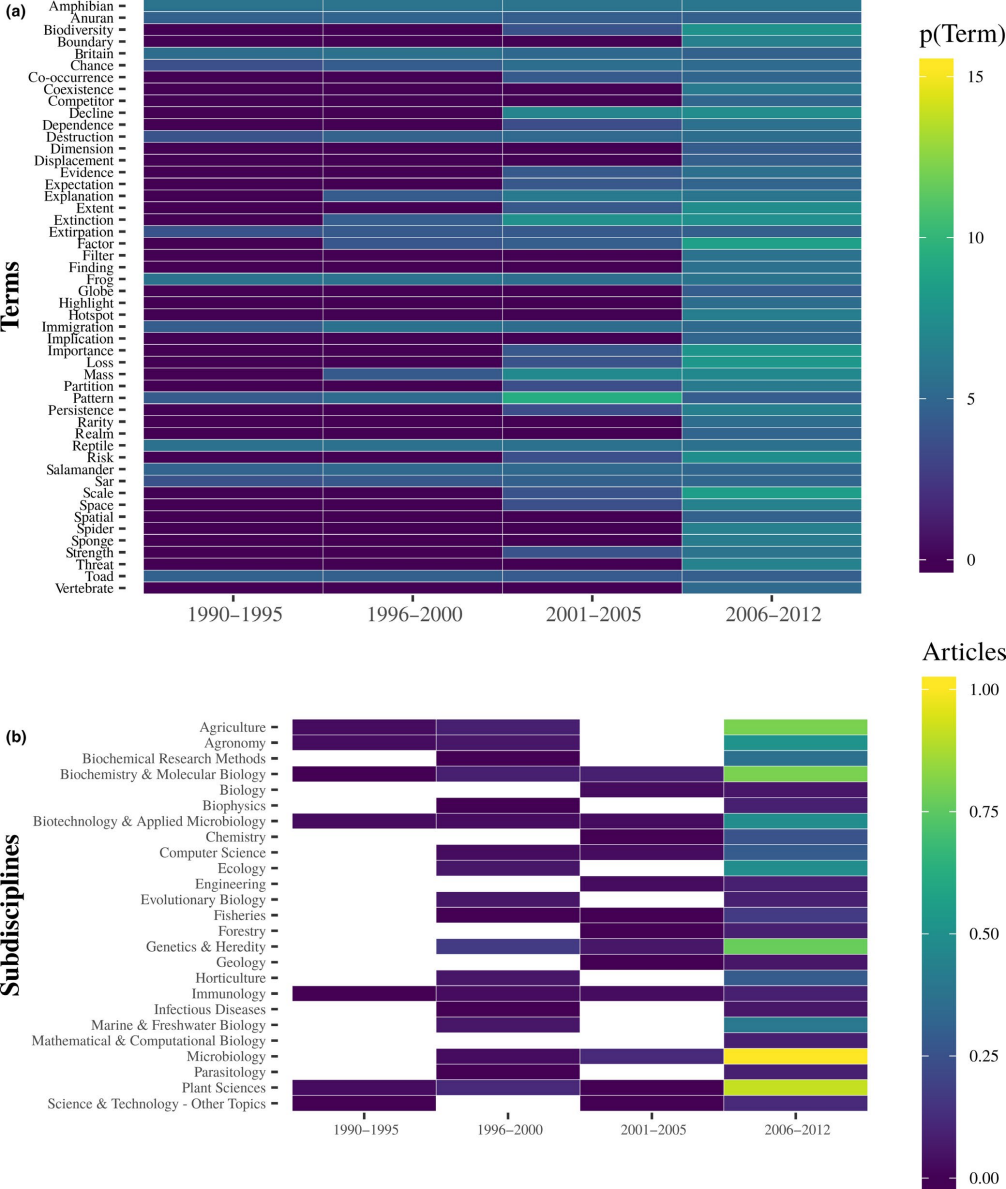
Temporal change in (a) the probability of highly ranked terms and (b) abundance of subdisciplines associated with “Concept 19,” a concept that addresses spatial patterns of biodiversity loss and their drivers and that became less interdisciplinary from 1990 to 2012. The top 50 terms from 2006 to 2012 (ranked by p(Term)) were selected. The top 25 ranked subdisciplines from 2006 to 2012 were selected, and the number of articles was scaled to facilitate comparisons with other concepts

We investigated temporal trends in interdisciplinarity of individual concepts in greater depth to better understand the overall pattern of temporal changes in interdisciplinarity in biodiversity science (for changes in terms and subdisciplines associated with each concept over time, see http://data.idiv.de/repo/Accelerating_interdisciplinarity_in_biodiversity_sciences/). Concepts that became increasingly interdisciplinary from 1990 to 2012 (Figure S4; http://data.idiv.de/repo/Accelerating_interdisciplinarity_in_biodiversity_sciences/) covered topics such as disease resistance in social insects (“Concept 8”; Figure 5) and the genetic isolation and divergence of plant populations (“Concept 28”). In contrast, concepts that become less interdisciplinary over time (Figure S4; http://data.idiv.de/repo/Accelerating_interdisciplinarity_in_biodiversity_sciences/) include ones focused on spatial patterns of biodiversity loss and their drivers (“Concept 19”; Figure 6) or species diversity indices (“Concept 18”). Concepts whose interdisciplinarity remained stable over time (gray lines, Figure S4) likely are used across a range of subdisciplines in biodiversity. For example, studies on biodiversity patterns and its drivers in protected areas and plantation forests maintained a similar level of disciplinarity over the study period (“Concept 10”; http://data.idiv.de/repo/Accelerating_interdisciplinarity_in_biodiversity_sciences/).

## 4 DISCUSSION

Biodiversity science is an emerging field replete with urgent, complex problems that cross disciplinary boundaries. Here, we present the first quantitative analysis of peer‐reviewed literature in this field and show that biodiversity science was as interdisciplinary—or less—in 2012 as it was in 1990.

We found that two proxies used to quantify interdisciplinarity in biodiversity science, concept and subdiscipline diversity, were either stable or declining between 1990 and 2012. The stable trend in concept and subdiscipline diversity (species richness; Figure 2) is likely attributable to the persistence of relatively rare concepts and subdisciplines, that is, those with fewer than 25 articles. Although rare, these concepts and subdisciplines contribute disproportionately to maintaining interdisciplinarity, a pattern that has also been found in the field of applied ecology (Staples, Dwyer, Wainwright, & Mayfield, 2019). In contrast, the decrease in the diversity of common concepts and subdisciplines (ENS_PIE_; Figure 2) appears to be driven by losses of concepts and subdisciplines that may have contributed previously to interdisciplinarity. More specifically, the significant decrease in temporal* β* concept diversity suggests that subdisciplines in biodiversity science have become more related over time (Figure 4). This pattern is consistent with the idea that biodiversity science is evolving into a mature discipline with a core set of broad concepts that are less specialized (Figure 3), while noncore concepts are being discarded (Graham & Dayton, 2002; Loreau, 2010). Drivers of this pattern likely include recent growth in collaborative research (Hoekman, Frenken, & Tijssen, 2010; Luukkonen, Persson, & Sivertsen, 1992; Tydecks, Jeschke, Wolf, Singer, & Tockner, 2018), which has benefited from greater institutional, financial, and infrastructural support within universities and funding programs that facilitate collaborations, for example, EU Horizon 2020, and synthesis centers (Baron et al., 2017; Ledford, 2015). Thus, it appears that collaborative research may have helped to build consensus within biodiversity science in terms of identifying its core concepts, but may not have increased its interdisciplinarity.

Because our study covers between the time period between 1990 and 2012, the conceptual homogenization observed in this study does not preclude increases in interdisciplinarity since 2012. The lack of an increase in most measures of interdisciplinarity in this study could be due to a combination of (a) a time lag in the translation from using similar concepts to substantive collaborations across biodiversity subdisciplines (detected by citations of interdisciplinary publications) and (b) higher barriers—particularly those related to research funding—when working interdisciplinarily (Bromham, Dinnage, & Hua, 2016; Jacobs & Frickel, 2009; Roy et al., 2013; Yegros‐Yegros, Rafols, & D'Este, 2015).

The marked variation in interdisciplinarity among concepts and subdisciplines in this study (Figure S4), which shows that biodiversity science is populated by a broad spectrum of approaches to science, is another potential explanation for the stable or decreasing overall trends in interdisciplinarity. All concepts and subdisciplines along the continuum of interdisciplinarity likely have the potential to contribute to advances in biodiversity science, but the tempo at which they do so may be influenced by their interdisciplinarity (Graham & Dayton, 2002) or research group size (Wu, Wang, & Evans, 2019). Highly interdisciplinary concepts may contribute to rapid or abrupt paradigm shifts, while more specialized, less interdisciplinary concepts may gradually change paradigms in biodiversity science.

Our analysis identified a number of concrete examples where greater interdisciplinarity in biodiversity science may contribute to enhancing scientific progress (Liu et al., 2007). One such example is that of disease resistance in social insects (“Concept 8”; Figure 5). Over time, this concept integrated expertise and knowledge from a diverse array of subdisciplines, such as genetics, evolutionary biology, biochemistry, and molecular biology, which provide mechanistic insights into large‐scale ecological patterns and contribute to identifying drivers of global declines in pollinators (Furst, McMahon, Osborne, Paxton, & Brown, 2014). Until recently, this concept had a narrower disciplinary scope, having evolved from the impacts of pollution on aquatic invertebrate communities (see terms from 1990 to 1995 in “Concept 8”; Figure 5). It is possible that the rapid emergence of specific threats to biodiversity within specific taxa, such as amphibians (Blaustein, Wake, & Sousa, 1994) and social bees (Furst et al., 2014), may have prompted researchers to seek synergistic collaborations with colleagues from other subdisciplines, such as molecular ecology and climate science. However, external factors that have catalyzed the evolution of these concepts toward greater interdisciplinarity could not be identified with our analysis and were beyond the scope of the methods used. Therefore, we cannot discard the possibility that growing public awareness of the biodiversity crisis may attract greater attention and funding initiatives may create incentives for researchers to superficially link their research to biodiversity‐related concepts by using certain buzzwords in publication titles and abstracts.

Similarly, our analysis identified concepts in biodiversity science that became less interdisciplinary during the study period. One of these concepts examines spatial patterns of biodiversity loss of specific taxa (e.g., amphibians, reptiles, and spiders) and their drivers (“Concept 19”; Figure 6) and is used across a range of both basic and applied subdisciplines, such as ecology, microbiology, molecular biology, and agriculture. Despite considerable interest in the impacts of land‐use and climate change on biodiversity at local and global scales (Newbold et al., 2015) and biodiversity loss on ecosystem functioning and services (Balvanera et al., 2006; Hooper et al., 2012), the decline in interdisciplinarity of this concept observed in this study possibly reflects an increase in specialization. Research on this concept may have become concentrated among an increasingly smaller number of subdisciplines in biodiversity science given its restricted taxonomic scope. Since 2012, the publication of comprehensive, open data sets containing the spatial and temporal distribution of species from multiple taxa such as ants, birds, plants, and reef fishes (Bruelheide et al., 2018; Dornelas et al., 2018; Economo, Narula, Friedman, Weiser, & Guénard, 2018; Edgar & Stuart‐Smith, 2014; Jetz, Thomas, Joy, Hartmann, & Mooers, 2012) may have altered the trajectory of interdisciplinarity for this concept. Indeed, the growing availability of biodiversity data (Allen, Folk, Soltis, Soltis, & Guralnick, 2019) may impact the interdisciplinarity of biodiversity science more broadly by overcoming at least one of the important barriers—access to information—that may have restricted its growth in the past.

## 5 CONCLUSIONS

In contrast to well‐established disciplines such as physics, mathematics, and medicine (Pan et al., 2012; Porter & Rafols, 2009), our results do not support the hypothesis that biodiversity science is becoming increasingly interdisciplinary. Rather, we observed either stable or declining concept or subdiscipline diversity. We also found a high degree of homogenization of concepts used by researchers in biodiversity science; researchers increasingly use similar scientific concepts as the foundation for their research, possibly reflecting recent growth in collaboration in biodiversity science (Tydecks et al., 2018). While greater interdisciplinarity is often invoked as a requirement for transformative research (Ledford, 2015; National Academy of Sciences, 2004), impactful science rarely occurs as a result of “one size fits all” mandates (Gravem et al., 2017; Wu et al., 2019). Greater interdisciplinarity in biodiversity science, therefore, may not be a necessary condition for researchers to produce high‐impact research, provided that it maintains a diverse array of approaches to research. Our findings thus raise the question: *Is the glass half full or half empty for biodiversity science?* The temporal trends in conceptual homogenization and interdisciplinarity revealed by our study may reflect that biodiversity science is a relatively young discipline that will become more interdisciplinary once better established and with growing support from initiatives such as the Intergovernmental Science‐Policy Platform on Biodiversity and Ecosystem Services (IPBES). Yet it may also reveal that its progress over twenty years toward addressing grand challenges facing humanity, such as climate change and food security, may have been impeded by the failure to adequately invest in societally relevant research that spans across concepts and disciplines.

## CONFLICT OF INTERESTS

The authors declare no competing interests.

## AUTHOR CONTRIBUTIONS

DC, MW, and CW conceived the project; JG and MH compiled and prepared the data; KH wrote the Java code for the LDA‐cDTM; DC analyzed the results of the LDA‐cDTM; DC and MW wrote the first draft of the manuscript; and all coauthors contributed substantially to revisions.

## Data Availability

Data supporting the findings of this study are available via DataDryad (DOI: https://doi.org/10.5061/dryad.04q1035). Code supporting the findings of this study is available via GitHub (https://github.com/idiv-biodiversity/BiodiversityLiterature).
