# Supplementary material for: Evolution of interdisciplinarity in biodiversity science
Source: Ecol Evol. 2019 Jun 10;9(12):6744–55. doi: 10.1002/ece3.5244 (PMC6640045; doi:10.1002/ece3.5244)
Supplement: Supplementary file 1 [file ECE3-9-6744-s001.pdf]

Supplementary Materials for

Evolution of interdisciplinarity in biodiversity science

Dylan Craven *, Marten Winter, Konstantin Hotzel, Jitendra Gaikwad, Nico Eisenhauer, Martin Hohmuth, Birgitta König-Ries, Christian Wirth

* Corresponding author. E-mail: dylan.craven@aya.yale.edu

This file includes:

Figs. S1 to S4

Supplementary Methods

Supplementary Note

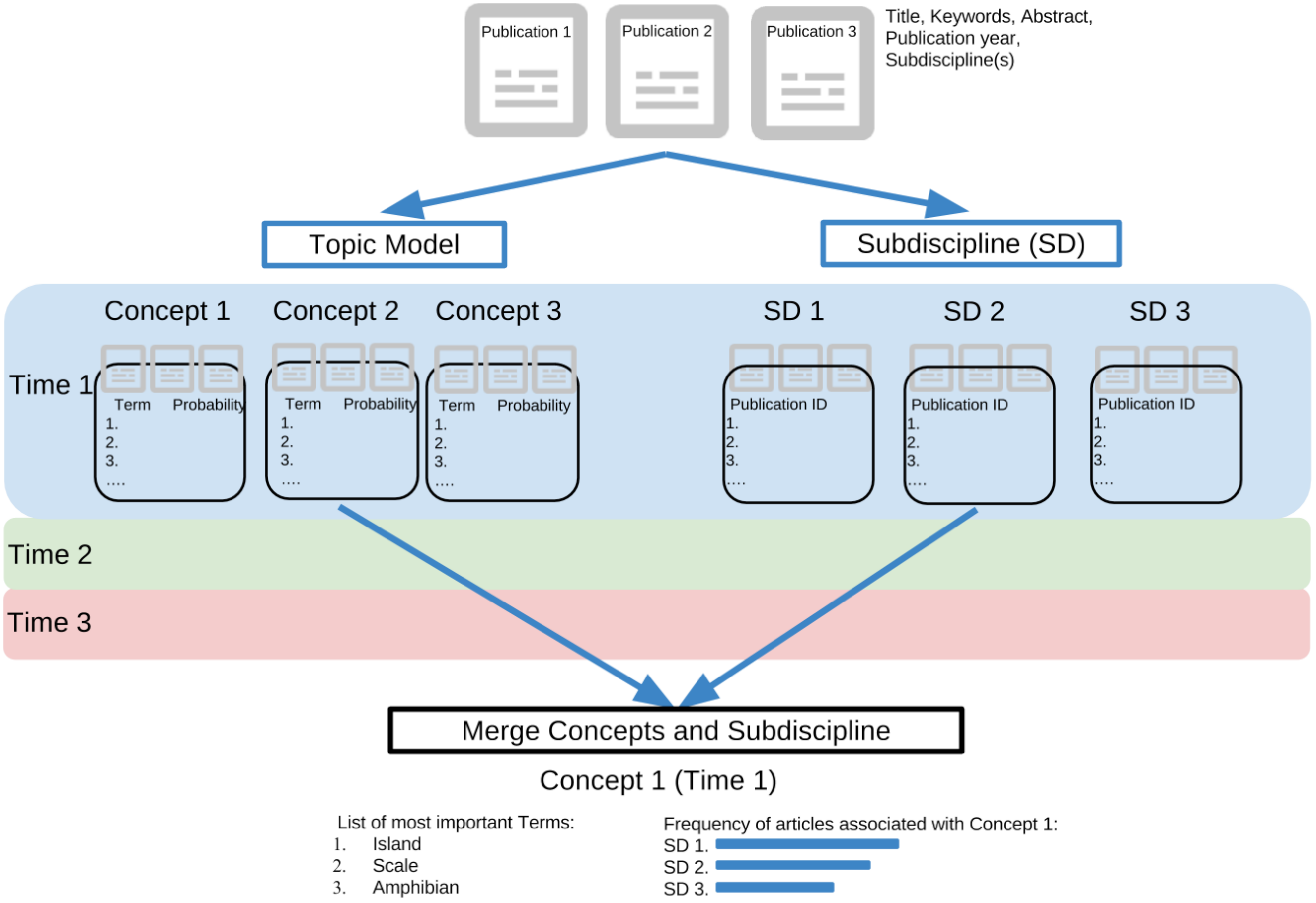

Fig. S1. Conceptual diagram illustrating the analysis workflow that integrated results from a continuous time dynamic topic model (LDA-cDTM) and subdisciplines (as designated by Thomson Reuters). Each article, therefore, could be associated with a concept and a subdiscipline (or multiple subdisciplines). Across all articles in a given time period, we extracted *i*) the most important terms associated with each concept and *ii*) the number of articles associated with each subdisciplines in a given concept.

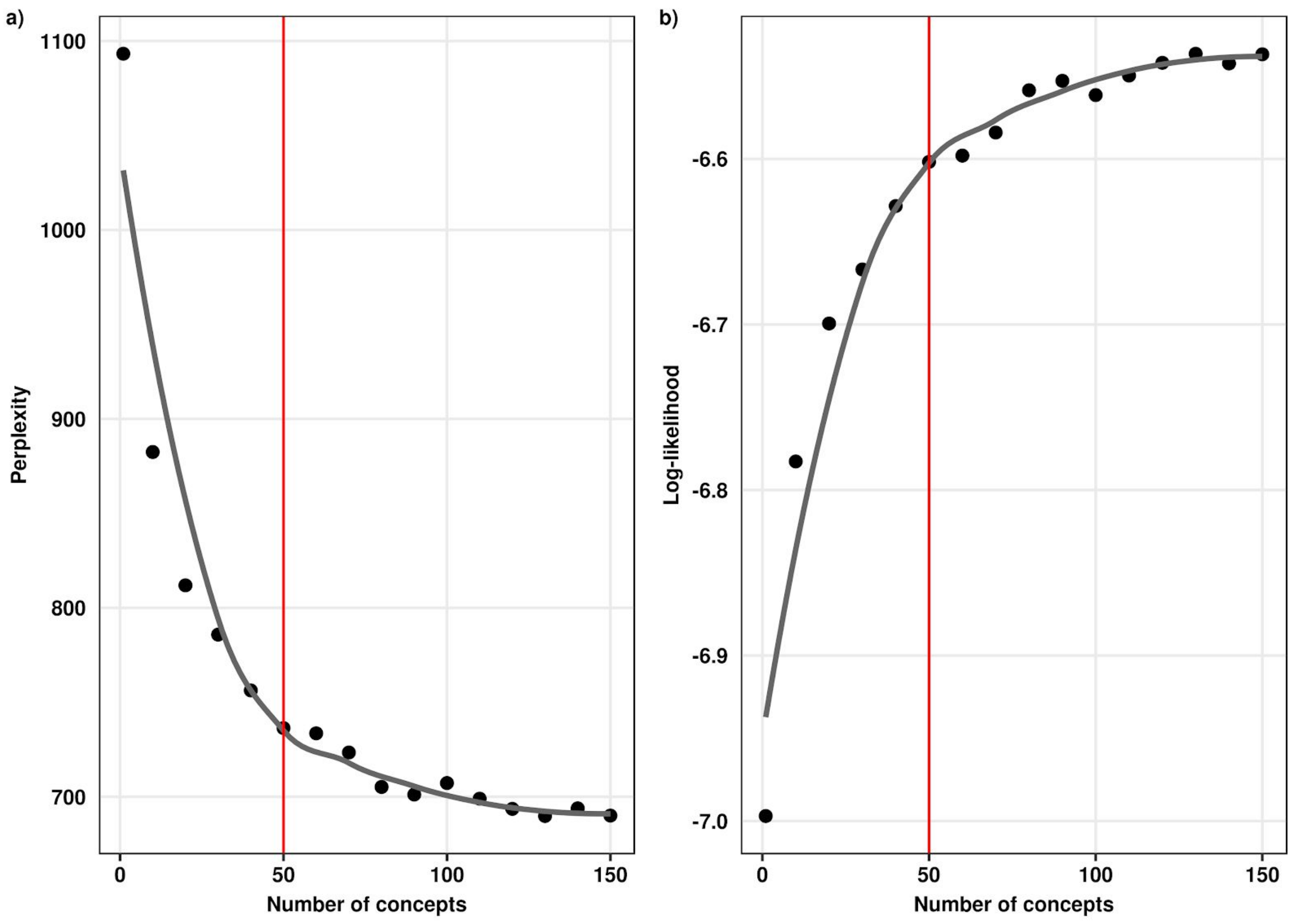

Fig. S2. Perplexity **(a)** and log-likelihood values **(b)** of continuous time dynamic topic models (LDA-cDTM) fitted across a range of numbers of concepts. The red line indicates the perplexity and log-likelihood values for the LDA-cDTM with 50 concepts.

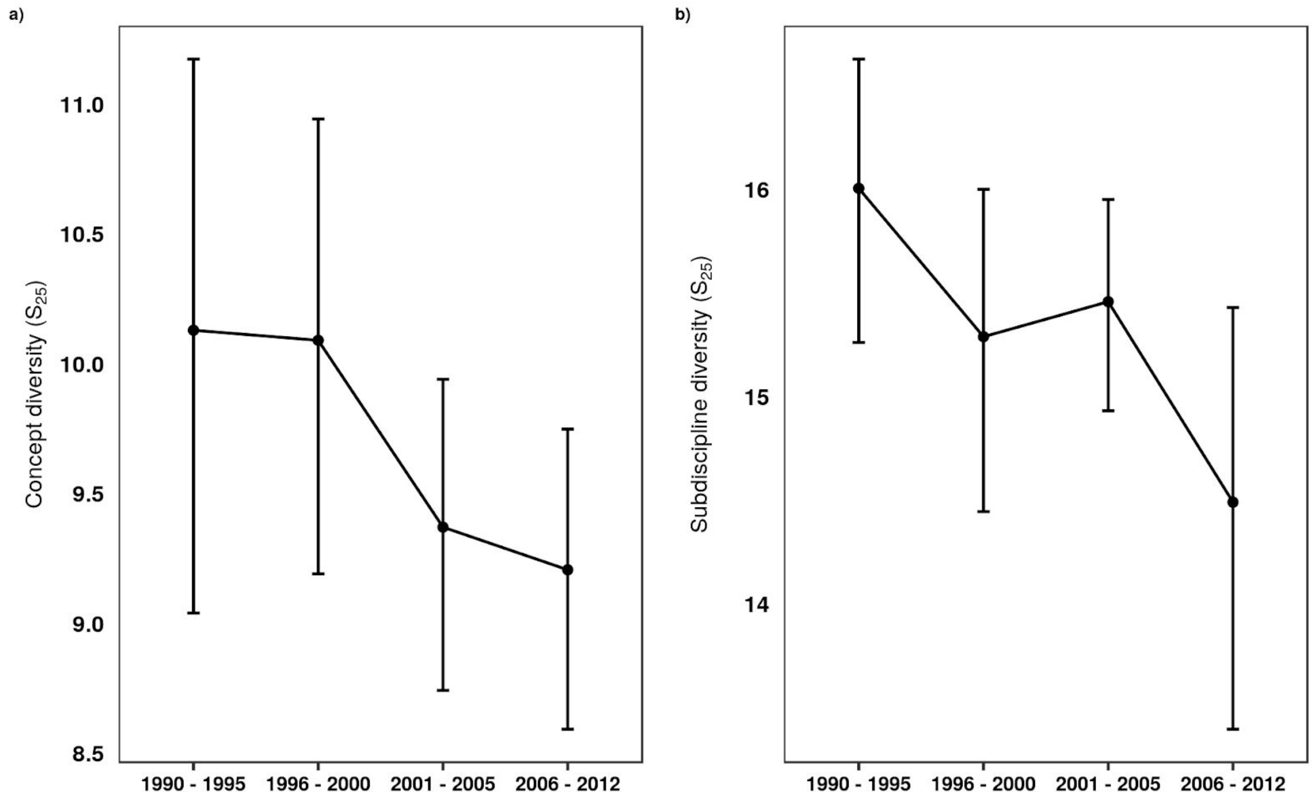

Fig. S3. Temporal changes in mean concept diversity (**a**) and mean subdiscipline diversity (**b**) between 1990 and 2012. Concept diversity and subdiscipline diversity were estimated using rarefied species richness (*n* = 25). Concept diversity is the number of concepts (identified by a continuous time dynamic topic model) associated with a subdiscipline in biodiversity science and subdiscipline diversity is the number of subdisciplines in biodiversity science associated with a concept (see Fig. 1). Whisker bars are bootstrapped 95% confidence intervals.

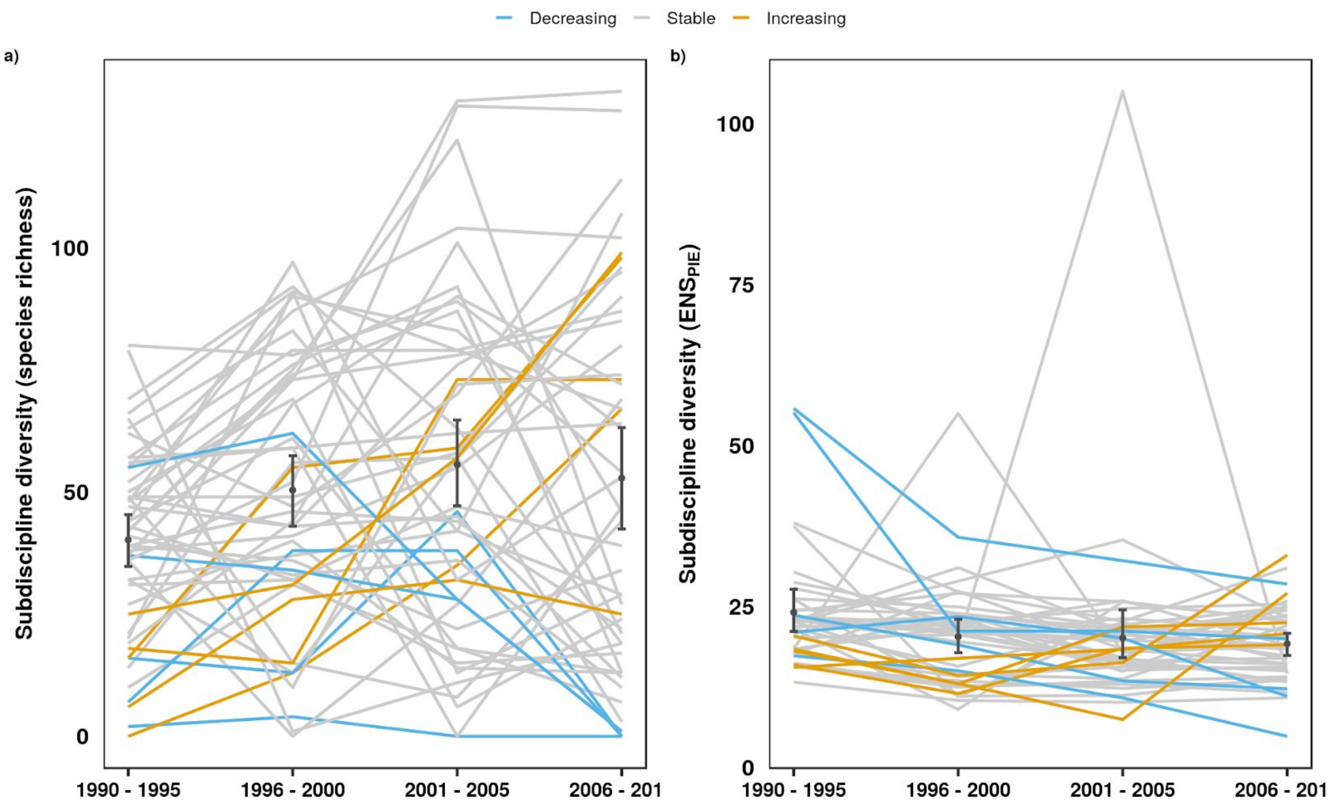

Fig. S4. Temporal changes in subdiscipline diversity between 1990 and 2012. Subdiscipline diversity was estimated using species richness and the effective number of species for the probability of interspecific encounter (ENS_{PIE}), respectively. The former highlights contributions of rare species, while the latter emphasizes contributions by dominant or highly abundant species. Subdiscipline diversity is the number of subdisciplines in biodiversity science associated with a concept, which was identified by a continuous time dynamic topic model. For ENS_{PIE} , abundance is the number of articles in a concept or subdiscipline. Orange lines represent concepts whose change in subdiscipline diversity (between 1990 and 2012) was classified as increasing over time, blue lines represent concepts whose change in subdiscipline diversity was classified as decreasing over time, and grey lines represent concepts whose change in subdiscipline diversity was classified as stable. Points and error bars are mean values and 95% bootstrapped confidence intervals for each time window. For an interactive version of this figure to see terms and subdisciplines associated with each concept in each time step, please follow this link:
http://data.idiv.de/repo/Accelerating_interdisciplinarity_in_biodiversity_sciences/.

Supplementary Methods.

We calculated the inverse document frequency (idf) for each term across all articles in the data set using the following formula:

$$idf = \log10 (N_d / N_t),$$

where N_d is the total number of articles in the dataset and N_t is the number of documents containing term t. We removed terms whose idf was greater than 3.04, i.e. terms that occurred infrequently across our data set. We removed these terms to improve the classification of texts into concepts by the continuous time dynamic topic model, but we did not test this directly. Rather, we assessed the sensitivity of the continuous time dynamic topic model across a range of numbers of concepts.

Supplementary Note.

Full query submitted to Web of Science (October 2013).

We queried the tagged Web of Science database for articles published between 1990 and 2012 containing the terms: 'biodiv*', 'species diversity', 'functional diversity', 'ecosystem diversity', and 'genetic diversity'.

To reduce false positives, we restricted our search to articles published in journals assigned to the following research areas:

Environmental Sciences & Ecology, Biodiversity Conservation, Physiology, Plant Sciences, Zoology, Computer Science, History & Philosophy Of Science, Genetics & Heredity, Chemistry, Microbiology, Gastroenterology & Hepatology, Biochemistry & Molecular Biology, Infectious Diseases, Biophysics, Agriculture, Demography, Marine & Freshwater Biology, Cell Biology, Pathology, Life Sciences & Biomedicine - Other Topics, Urban Studies, Science & Technology - Other Topics, Public, Environmental & Occupational Health, Forestry, Energy Fuels, Mathematics, Geology, Reproductive Biology, Cultural Studies, Geography, Anthropology, Meteorology & Atmospheric Sciences, Evolutionary Biology, Immunology, Entomology, Virology, Physical Geography, Mycology, Developmental Biology, Biotechnology & Applied Microbiology, Tropical Medicine, Oceanography, Remote Sensing, Anatomy & Morphology, Oncology, Toxicology, Microscopy, Archaeology, Arts & Humanities - Other Topics, Fisheries, Mathematical & Computational Biology, Behavioral Sciences, Parasitology, Water Resources, Imaging Science & Photographic Technology, Paleontology, Sociology
